# Leptin Signaling Contributes to Aromatase Inhibitor Resistant Breast Cancer Cell Growth and Activation of Macrophages

**DOI:** 10.3390/biom10040543

**Published:** 2020-04-03

**Authors:** Luca Gelsomino, Cinzia Giordano, Giusi La Camera, Diego Sisci, Stefania Marsico, Antonella Campana, Roberta Tarallo, Antonio Rinaldi, Suzanne Fuqua, Antonella Leggio, Fedora Grande, Daniela Bonofiglio, Sebastiano Andò, Ines Barone, Stefania Catalano

**Affiliations:** 1 Department of Pharmacy, Health and Nutritional Sciences, Via P Bucci, University of Calabria, 87036 Arcavacata di Rende (CS), Italy; luca.gelsomino@unical.it (L.G.); cinzia.giordano@unical.it (C.G.); giusylacamera93@gmail.com (G.L.C.); dsisci@unical.it (D.S.); stefania.marsico@unical.it (S.M.); anto88c@gmail.com (A.C.); antonella.leggio@unical.it (A.L.); fedora.grande@unical.it (F.G.); daniela.bonofiglio@unical.it (D.B.); sebastiano.ando@unical.it (S.A.); 2 Laboratory of Molecular Medicine and Genomics, Department of Medicine, Surgery and Dentistry “Scuola Medica Salernitana”, University of Salerno, 84081 Baronissi (SA), Italy; rtarallo@unisa.it (R.T.); a.rinaldi@izs.it (A.R.); 3 Lester and Sue Smith Breast Center, Baylor College of Medicine, One Baylor Plaza, MS: 600 N1220.01 Alkek Building, Houston, TX 77030, USA; sfuqua@bcm.tmc.edu

**Keywords:** breast cancer, endocrine resistance, leptin, macrophages, obesity

## Abstract

Obesity represents a risk factor for breast cancer development and therapy resistance, but the molecular players underling these links are unclear. Here, we identify a role for the obesity-cytokine leptin in sustaining aromatase inhibitor (AI) resistant growth and progression in breast cancer. Using as experimental models MCF-7 breast cancer cells surviving long-term treatment with the AI anastrozole (AnaR) and Ana-sensitive counterparts, we found that AnaR cells expressed higher levels of leptin and its receptors (ObR) along with a constitutive activation of downstream effectors. Accordingly, leptin signaling inhibition reduced only AnaR cell growth and motility, highlighting the existence of an autocrine loop in mechanisms governing drug-resistant phenotypes. In agreement with ObR overexpression, increasing doses of leptin were able to stimulate to a greater extent growth and migration in AnaR than sensitive cells. Moreover, leptin contributed to enhanced crosstalk between AnaR cells and macrophages within the tumor microenvironment. Indeed, AnaR, through leptin secretion, modulated macrophage profiles and increased macrophage motility through CXCR4 signaling, as evidenced by RNA-sequencing, real-time PCR, and immunoblotting. Reciprocally, activated macrophages increased AnaR cell growth and motility in coculture systems. In conclusion, acquired AI resistance is accompanied by the development of a leptin-driven phenotype, highlighting the potential clinical benefit of targeting this cytokine network in hormone-resistant breast cancers, especially in obese women.

## 1. Introduction

At clinical presentation, approximately three-quarters of all primary breast tumors are categorized as estrogen receptor (ER) α positive, endorsing the deprivation of estrogen signaling through endocrine-targeted therapy (i.e., aromatase inhibitors (Ais)) as the mainstay of treatment both in the adjuvant and recurrent settings. However, despite the obvious benefits, many patients will exhibit initial or acquired resistant disease associated with reduced survival. A number of researchers have generated different cell line models with the aim of understanding the mechanisms of such AI resistance on the molecular level and developing avenues to overcome it [1,2,3,4,5]. The most attractive hypothesis is that resistance implies a selection process that involves the activation of alternative regulatory survival pathways allowing cancer to recur, although estrogen production is suppressed by AIs [6,7]. In particular, aberrant crosstalk among growth factor signaling pathways, including epidermal growth factor receptor (EGFR) and human epidermal growth factor receptor 2 (HER2), and the ER signaling converging in the activation of downstream RAF/MEK/ERK and PI3K/Akt/mTOR pathways has been shown to be crucial in driving cell survival and proliferation in many patients progressing on endocrine treatment [6,8]. Therefore, a few agents targeting these signaling pathways have been explored in current clinical trials, but results have been somewhat unsatisfactory [7], highlighting the unmet need of elucidating other dynamic changes that enable breast cancer cells to escape over the course of treatment.

Current studies suggest that the behavior of breast cancer cells is strictly dependent on adequate extrinsic factors, which may serve as a driving force in breast carcinogenesis [9]. Potential cell-extrinsic host milieu may include several obesity-related factors that include components of the adipocyte secretome, such as hormones (e.g., estrogens), growth factors (e.g., insulin-like growth factor I), and adipokines (e.g., leptin). Evidence suggests that the obese setting provides a unique adipose tissue microenvironment that, in association with systemic endocrine modifications, promotes breast tumor initiation, primary growth, invasion, and metastatic progression [10,11]. Indeed, obesity has been associated with poor breast cancer outcomes, including larger tumor size, lymph node positivity, regional/distant stage, reduced therapy effectiveness, and high rate of mortality, especially in post-menopausal ERα-positive subgroups [12,13,14]. In this regard, a growing body of data have outlined the role of the obesity cytokine leptin-mediated pathways in mammary tumorogenesis, proposing this adipokine as a key member of the molecular network in obesity. Indeed, both leptin and its receptors (ObR) are found to be overexpressed in breast carcinomas, where they correlate with higher grade, distant metastasis, and poor prognosis [15,16,17,18,19,20]. The leptin/ObR axis through the activation of JAK2/STAT3, MEK, and PI3K/Akt signalings intimately controls different cellular activities, including mitogenesis, survival, transformation, migration, and invasion of breast cancer cells [14,21,22]. In addition to its direct action, other authors and we have demonstrated that leptin can modulate breast cancer biology by interacting with different signaling molecules, such as estrogen and growth factors [16,23,24,25,26,27,28]. Of note, leptin is able to shape the tumor microenvironment by inducing multiple concurrent events, for example, a promotion of angiogenesis and a sustained recruitment of monocytes and macrophages, which in turn secrete VEGF (vascular endothelial growth factor) and proinflammatory cytokines [29,30,31]. However, only a few studies have indicated a role of this adipokine in endocrine resistance, such as tamoxifen resistance [26,32,33,34,35,36,37], and so far, its involvement in AI therapy resistance has not yet been investigated. Here, we present data showing the involvement of the leptin-mediated signaling pathway in the development of AI therapy resistance, thereby suggesting how blocking leptin signaling could be further exploited for clinical utility in breast cancer resistant settings, especially for obese patients.

## 2. Materials and Methods

### 2.1. Reagents, Antibodies, and Plasmids

The following reagents and antibodies were used: leptin and zeocin from Thermo Fisher Scientific (Waltham, MA, USA), anastrozole, and 4-androstene-3,17-dione from Sigma–Aldrich (Milan, IT, Italy); human anti-aromatase antibody from Serotec (Oxford, UK, Britain); human anti-ERα, anti-glyceraldehyde-3- phosphate dehydrogenase (GAPDH), anti-PR, anti-ObR, anti-Akt, anti-pAkt^Ser437^, and anti-CXCR4 antibodies from Santa Cruz Biotechnology (Dallas, TX, USA); human anti-JAK2, anti-pJAK2^Tyr1007/1008^, anti-STAT3, anti-pSTAT3^Tyr705^, anti-MAPK, anti-pMAPK^Thr202/Tyr204^ antibodies from Cell Signaling Technology (Denver, MA, USA). XETL plasmid, containing an estrogen-responsive element, was provided by Dr. Picard (University of Geneva, Geneva, Switzerland).

### 2.2. Cell Culture

MCF-7 aro cells stably expressing the aromatase enzyme were generated and maintained as previously described [38]. Anastrozole-resistant MCF-7 cells (AnaR) cells were generated by culturing MCF-7 aro cells in MEM with 10% FBS, 1% L-glutamine, 1% Eagle’s nonessential amino acids, 1 mg/mL penicillin/streptomycin, zeocin 0.2 mg/mL, and anastrozole 1 μM. Cells were routinely maintained in 1 μM anastrozole for longer than one year. The murine macrophage RAW 264.7 cell line was cultured in DMEM medium containing 10% FBS, 1% L-glutamine, 1% Eagle’s nonessential amino acids, and 1 mg/mL penicillin–streptomycin.

All cell lines, stored and authenticated following suppliers, were used within six months after frozen aliquot resuscitations and were regularly tested for mycoplasma-negativity (MycoAlert Mycoplasma Detection Assay, Lonza, Basilea, CH, Switzerland).

### 2.3. Cell Proliferation Assays

MTT assays: Cell viability was determined by using 3-(4,5-dimethylthiazol-2-yl)-2,5-diphenyltetrazolium bromide (MTT, Sigma–Aldrich) reagent as previously described [39].

Soft agar growth assays: Soft-agar anchorage-independent growth assays were performed as previously described [40]. Briefly, cells (25000/well) were plated in 4 mL of 0.35% agarose with 5% charcoal-stripped FBS in phenol red-free media, in a 0.7% agarose base in six-well plates. Two days after plating, a medium containing control vehicle or treatments was added to the top layer, and the medium was replaced every two days. After 14 days, 150 μL of MTT was added to each well and allowed to incubate at 37 °C for 4 h. Plates were then placed in 4 °C overnight, and colonies >50 μm diameter were counted.

Data represent three independent experiments performed in triplicate.

### 2.4. ERE-Luciferase Reporter Assay

Cells were transiently transfected using the FuGENE 6 reagent (Promega, Madison, WI, USA) as recommended by the manufactures with XETL reporter plasmid (0.5 μg/well) and TK Renilla luciferase plasmid (25 ng/well). After 6 h, cells were treated as indicated for 18-24 h. The firefly and Renilla luciferase activities were measured using a dual luciferase kit (Promega), as previously described [41]. The firefly luciferase data for each sample were normalized on the basis of transfection efficiency measured by Renilla luciferase activity and reported as fold.

### 2.5. Aromatase Activity Assays

The aromatase activity was measured by the tritiated water release assay using 0.5 μM [1β-^3^H]-androst-4-ene-3,17-dione as a substrate (Perkin Elmer, Waltham, MA, USA). The incubations were performed at 37 °C for 2 h. The results obtained were expressed as picomole/hour and normalized to milligram of protein (pmol/h/mg protein).

### 2.6. Real-Time PCR Assays

Gene expression was evaluated by real-time reverse transcription (RT)-PCR, using SYBR Green Universal PCR Master Mix (Bio-Rad, Hercules, CA, USA). Each sample was normalized on 18S or GAPDH content, depending on its human or mouse origin, and relative gene expression levels were calculated as previously described [42].

Primers used are listed in Supplementary Table S1.

### 2.7. Immunoblot Analysis

Equal amounts of cell extracts were resolved by SDS-PAGE, as described in [43]. Immunoblots show a single representative of three separate experiments. The bands of interest were quantified by the Scion Image laser densitometry scanning program, and standard deviations along with associated *p*-values for the replicates were calculated by GraphPad-Prism7 software (GraphPad Inc., San Diego, CA, USA).

### 2.8. Leptin Measurement by Enzyme-Linked Immunosorbent Assay (ELISA)

Human leptin was measured in breast cancer cell media by using the LDN leptin ELISA Kit based on the manufacturer’s protocol (Labor Diagnostika Nord, Nordhorn, DE, Germany). Results are presented as nanograms per milliliter of media (ng/mL).

### 2.9. Wound-Healing Assays

Cell migration was assessed, and wound closure was monitored over 24 h, as previously reported [44]. Images represent one of three independent experiments (10× magnification) (OLIMPUS-BX51 microscope). The rate of wound-healing was quantified, as described in [45].

### 2.10. Migration/Chemiotaxis Assays

Cells treated as indicated were placed in the upper compartment of a Boyden chamber (8 μm membranes; Corning Costar, Corning, NY, USA). The bottom well contained the specific chemoattractant as reported in the different experimental conditions. After 12–18 h, migrated cells were fixed and stained with Coomassie brilliant blue. Migration was quantified by viewing 5 separate fields per membrane at 20× magnifications and expressed as the mean number of migrated cells.

### 2.11. Conditioned Medium and Coculture Systems

MCF-7 aro and AnaR cells were incubated with red phenol-free media supplemented with 5% charcoal-stripped FBS (48 h). Conditioned media (CM) were collected, centrifuged to remove cellular debris, and used in the respective experiments. In another set of experiments, RAW 264.7 cells were incubated with MCF-7 aro and AnaR CM for 24 h. Then, cells were washed twice and cultured with 5% charcoal-stripped serum medium for 48 h. CMs were collected, centrifuged, and used in co-culture experiments with breast cancer cells.

### 2.12. Leptin-Immunodepleted Conditioned Media

Protein G-agarose beads were incubated with anti-leptin or IgG antibodies for 3 h at 4 °C. Antibody-beads complexes were incubated with AnaR cell-derived conditioned medium overnight and centrifuged. Leptin immunodepletion was verified by ELISA.

### 2.13. RNA Sequencing Analysis

Total RNA was extracted from RAW 264.7 cells treated with MCF-7 aro CM or AnaR CM, and libraries were prepared and sequenced as described [44]. RNA sequencing data were deposited in the EBI ArrayExpress database (http://www.ebi.ac.uk/arrayexpress) with Accession Number E-MTAB-8843. Sequencing reads (50–60 million reads/sample on average) were trimmed, quality filtered, and aligned, including junction-spanning reads back, to the mouse genome (Mus musculus Ensemble NCBIM37) using Tophat v.2.0.10 (Center for Bioinformatics and Computational Biology, University of Maryland, College Park, MD, USA) [46]. HTSeq [47] was used to compute read counts across each gene, which were then used as input to R package DESeq2 [48] to normalize read counts for library size and dispersion followed by tests for differential gene expression. Significant differentially expressed genes were determined using false discovery rate (FDR) cutoff ≤ 0.05 and at least 1.5-fold change between conditions. Functional analyses were performed with the Ingenuity Pathway Analysis suit (Ingenuity Systems).

### 2.14. Statistical Analysis

Each datum point represents the mean ± S.D. of three different experiments. Data were analyzed for statistical significance using a two-tailed Student’s *t*-test, and the GraphPad-Prism7 software program.

## 3. Results

### 3.1. Anastrozole-Resistant (AnaR) Cells Exhibit Increased Activation of Leptin Signaling Pathway

**Figure 1:**
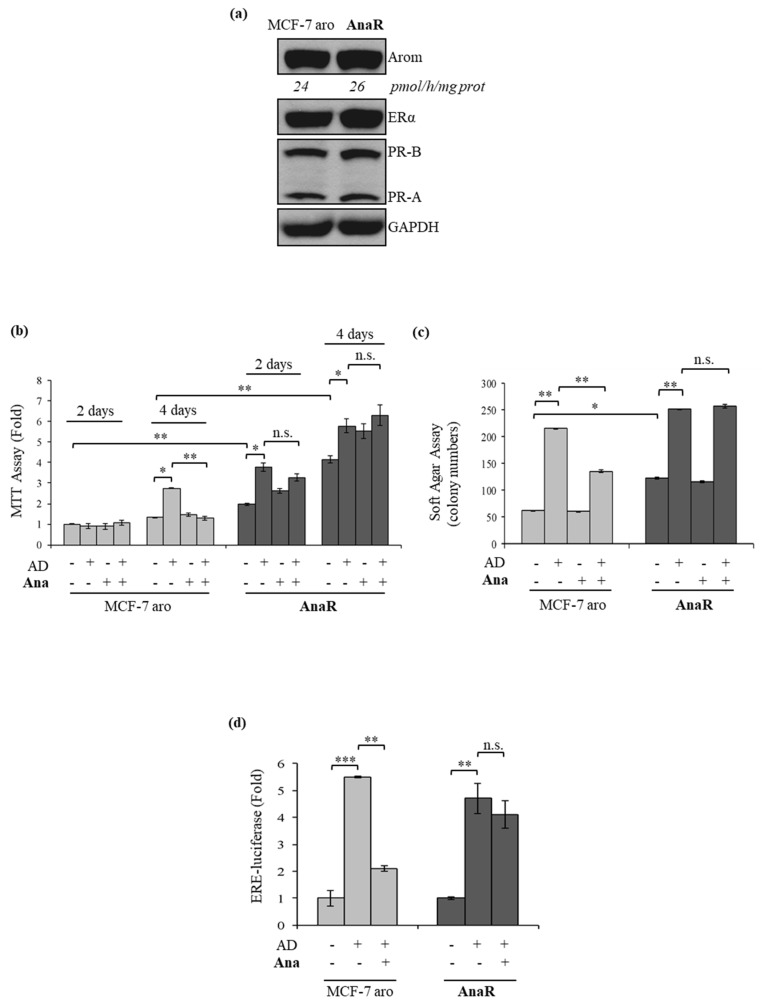
Characterization of anastrozole-resistant (AnaR) breast cancer cells. (**a**) Immunoblotting for expression of aromatase, estrogen receptor (ER) α, progesterone receptors (PR-B and PR-A) in MCF-7 aro, and AnaR breast cancer cells. Anti-glyceraldehyde-3- phosphate dehydrogenase (GAPDH) was used as a control for equal loading and transfer. Numbers below blots represent aromatase activity values. (**b**) Anchorage-dependent 3-(4,5-dimethylthiazol-2-yl)-2,5-diphenyltetrazolium bromide (MTT) growth assays in MCF-7 aro and AnaR cells treated with the aromatase substrate 4-androstene-3,17-dione (AD, 10 nM/L), in the presence or not of anastrozole (Ana, 1 μM) for 2 and 4 days. (**c**) Soft agar growth assays in cells stimulated for 14 days with AD and/or Ana. (**d**) Cells were transiently transfected with ERE-luciferase reporter gene and, after 6h, were treated with AD alone or in combination with Ana for 18-24h. n.s., non-significant, * *p* < 0.05; ** *p* < 0.005; *** *p* < 0.0005.

To evaluate whether leptin signaling may have a role in driving aromatase inhibitor resistance, as an adequate aromatase/estrogen receptor (ER) α positive model system to study aromatase inhibitor response, we generated a therapy refractory cancer cell line by culturing parental human MCF-7 breast cancer cells stably transfected with an aromatase expression vector (MCF-7 aro) [38] continuously in the presence of the non-steroidal AI anastrozole (termed as AnaR cells). As shown by immunoblotting analysis (Figure 1a), these cells did not show any major changes in protein expression and activity of aromatase as well as in the levels of estrogen and progesterone receptors. To test for AI resistance (AI^R^), we examined the effects of anastrozole in different in vitro assays. Anchorage-independent MTT assay revealed that the growth of MCF-7 aro cells was significantly enhanced after four days of treatment with androstenedione (AD), which aromatase converts to estrogen, and as expected Ana completely blocked this stimulation (Figure 1b). In contrast, in AnaR cells, AD enhanced growth earlier, and exposure to Ana was unable to inhibit AD-induced effects. To extend the MTT data, we performed anchorage-independent soft agar growth assays (Figure 1c). As expected, AD treatment increased colony numbers and treatment with Ana completely abrogated AD-stimulated growth in parental Ana-sensitive cells. In AnaR cells, colony number was further stimulated by AD, and Ana was unable to inhibit AD-induced colony formation. In both experiments, the basal growth of AnaR cells was higher compared to that of MCF-7 aro cells indicating a more aggressive phenotype of resistant cells. Concomitantly, in ERE-luciferase reporter assays treatment with Ana had no effect on AD-induced ERα transcriptional activity in the resistant cell line that we have generated (Figure 1d), confirming the AI^R^ phenotype.

**Figure 2:**
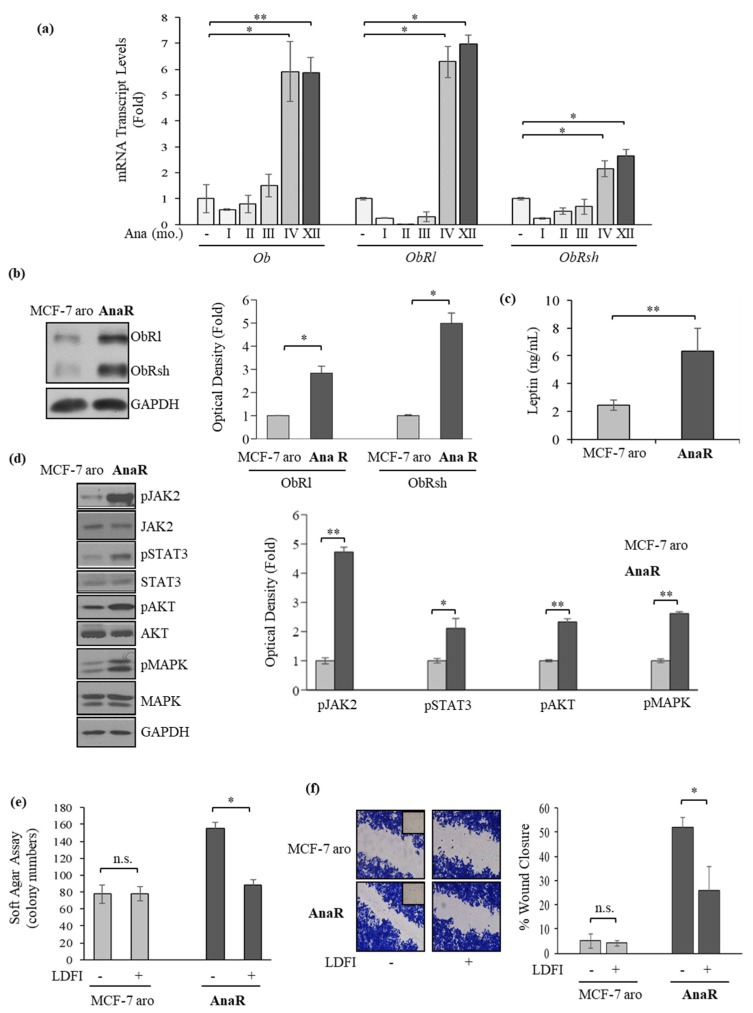
Increased leptin signaling activation in AnaR breast cancer cells. (**a**) Quantitative real-time PCR for mRNA expression of leptin *(Ob)*, the long (*ObRl*), and short (*ObRsh*) isoforms of its receptors in MCF-7 aro and AnaR cells along the different months (mo.) of treatment with Ana (1 µM). (**b**) Immunoblotting for expression of ObRl and ObRsh in MCF-7 aro and AnaR cells. GAPDH was used as a control for equal loading and transfer. The histograms represent the mean ± S.D. of three separate experiments in which band intensities were evaluated in terms of optical density arbitrary units and expressed as fold over MCF-7 aro. (**c**) Enzyme-linked immunosorbent assay (ELISA) for leptin secretion in MCF-7 aro and AnaR cells. (**d**) Immunoblotting for phosphorylation levels (*p*) of JAK2, STAT3, AKT, and MAPK along with total non-phosphorylated proteins in MCF-7 aro and AnaR cells. GAPDH was used as a control for equal loading and transfer. The histograms represent the mean ± S.D. of three separate experiments in which band intensities were evaluated in terms of optical density arbitrary units and expressed as fold over MCF-7 aro. (**e**) Soft agar growth assays in MCF-7 aro and AnaR cells treated with vehicle (-) or the peptide LDFI (1 µM). (**f**) Wound healing assays in cells exposed to vehicle (-) or the peptide LDFI (1 µM). Inset, time 0. Pictures are representative of three independent experiments. The histograms represent the relative percentage of wound closure calculated by image analysis using ImageJ software. n.s., non-significant, * *p* < 0.05; ** *p* < 0.005.

First, we evaluated using real-time PCR specific transcript levels of leptin and the long and short leptin receptor isoforms (ObRl and ObRsh) in MCF-7 aro at the start of treatment with Ana and after different time points until cells acquired the resistant phenotype (~4th month). We observed that the prolonged treatment with Ana induced a progressive phenotypic shift characterized after the 4th month of treatment by increased mRNA expression of leptin and its receptors, persisting over time (Figure 2a). The increase in both ObRl and ObRsh was then confirmed by evaluating protein levels using immunoblotting in AnaR compared to MCF-7 aro cells (Figure 2b), whereas ELISA measurement in breast cancer cell-derived conditioned media showed that AnaR cells exhibited a 2.5 fold higher leptin secretion than MCF-7 aro cells (Figure 2c). These results imply that an enhanced leptin autocrine feedback loop may exist in AnaR cells. Indeed, resistant cells exhibited increased constitutive phosphorylation levels of the leptin downstream effectors JAK2, STAT3, AKT, and MAPK (Figure 2d). Accordingly, treatment with the peptide LDFI, a small peptide of the wild type sequence of leptin binding site I, that we have recently demonstrated to specifically inhibit both in vitro and in vivo the leptin signaling pathway [49], significantly reduced the increased basal anchorage-independent growth (Figure 2e) and motility (Figure 2f) in AnaR cells, indicating a selective dependency on leptin signaling for this cell line.

### 3.2. Anastrozole-Resistant Breast Cancer Cells Show Leptin Hypersensitivity

**Figure 3:**
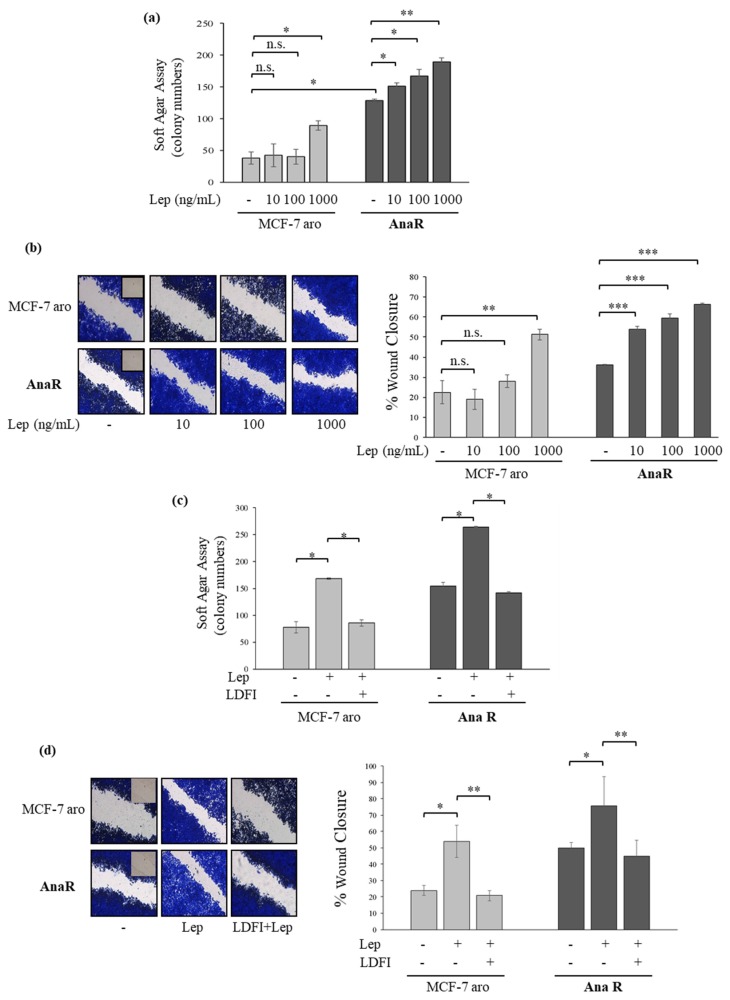
Leptin hypersensitivity in AnaR cells. (**a**) Soft agar growth assays in MCF-7 aro and AnaR breast cancer cells stimulated for 14 days with vehicle (-) or increasing doses of leptin. (**b**) Wound healing assays in cells exposed to vehicle (-) or leptin (Lep) as indicated. Inset, time 0. Pictures are representative of three independent experiments. The histograms represent the relative percentage of wound closure calculated by image analysis using ImageJ software. (**c**) Soft agar growth assays in MCF-7 aro and AnaR cells stimulated for 14 days with vehicle (-), leptin (1000 ng/mL), and the peptide LDFI (1 µM). (**d**) Wound healing assays in cells treated with vehicle (-), Leptin (1000 ng/mL), and the peptide LDFI (1 µM). Inset, time 0. Pictures are representative of three independent experiments. The histograms represent the relative percentage of wound closure calculated by image analysis using ImageJ software. n.s., non-significant, * *p* < 0.05; ** *p* < 0.005; *** *p* < 0.0005.

The adipocyte-derived leptin, whose synthesis and plasma levels increase in parallel to total adipose tissue mass, has an important role in promoting breast cancer progression. Thus, we also evaluated the effects of exogenous leptin stimulation on growth in our anastrozole-resistant cell models (Figure 3a). In a dose-response study, we observed that low concentrations of leptin (10 and 100 ng/mL) were able to increase colony numbers in anchorage-independent growth assays only in AnaR cells. In addition, treatment with leptin at 1000 ng/mL enhanced cell growth in both sensitive and resistant cells, although to a greater extent in AnaR cells. We also evaluated the ability of increasing doses of leptin to influence cell migration in wound-healing scratch assays (Figure 3b). The resistant cells moved more rapidly at the front of cell migration to close the gap compared with MCF-7 aro cells. Leptin treatments at 1000 ng/mL promoted cell motility in both cells, but at a higher extent in resistant cells. Interestingly, leptin at 10 and 100 ng/mL stimulated migration only in AnaR cells. The increase in colony numbers and migration induced by leptin was reversed by the peptide LDFI (Figure 3c,d, respectively). Therefore, increased leptin sensitivity may likely support AI^R^ in breast cancer cells.

### 3.3. Enhanced Recruitment and Protumor Activation of Macrophages from AnaR Cells throughLeptin Signaling

**Figure 4:**
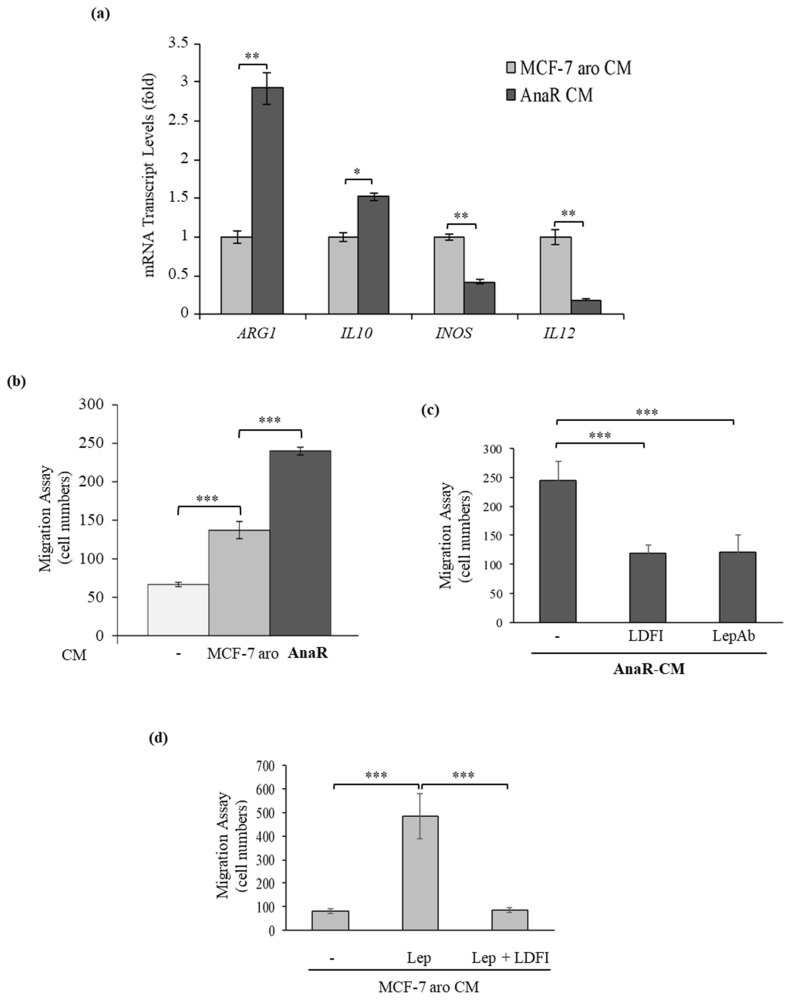
AnaR cells recruit and activate macrophages through leptin signaling. (**a**) Quantitative real-time PCR for mRNA expression of *ARG1* (Arginase 1), *IL10* (Interleukin-10), markers of pro-tumor macrophages and *IL12* (Interleukin-12), *INOS* (inducible Nitric Oxide Synthase), markers of tumor-inhibitory macrophages, in RAW 264.7 cells after incubation with conditioned medium (CM) derived from MCF-7 aro or AnaR cells. (**b**) Transmigration assay in RAW 264.7 cells using as a chemoattractant charcoal-stripped serum media (-) or conditioned medium (CM) derived from MCF-7 aro or from AnaR cells. (**c**) Transmigration assay in RAW 264.7 cells treated or not with the peptide LDFI (1 µM) using as chemoattractant AnaR-CM or AnaR-CM in which leptin was immunodepleted by incubation with a mouse monoclonal specific antibody against leptin (LepAb). (**d**) Transmigration assay in RAW 264.7 cells treated with vehicle (-), leptin (1000 ng/mL) alone or in the presence of the peptide LDFI (1 µM) using as a chemoattractant MCF-7 aro CM. * *p* < 0.05; ** *p* < 0.005; *** *p* < 0.0005.

It is well known that inflammation is involved in almost all tumor processes. Notably, leptin, in addition to its direct effects on breast cancer epithelial cells, has been demonstrated to stimulate the function of macrophages, one of the most abundant and critical immune cell types within the breast tumor microenvironment [22,50,51,52]. Thus, we investigated whether an enhanced production of leptin from AnaR cells may impact the macrophage phenotype to support malignant progression further. For this aim, first, the murine macrophage cell line RAW 264.7 was exposed to conditioned medium (CM) from Ana-sensitive or resistant cells to evaluate the expression of a number of genes associated with macrophage phenotype (Figure 4a). Real-time PCR analysis revealed that exposure of macrophages to AnaR-CM leads to an increase in the expression levels of the immunosuppressive markers Arginase 1 (*ARG1*) and interleukin-10 (*IL10*), concomitant with a decrease in the expression levels of the tumor inhibitor markers inducible NO synthase (*INOS*) and *IL12*, as typically observed in tumor-associated macrophages (TAMs). However, no differences in the levels of transforming growth factor beta (*TGF-β*) and *SMAD3* were detected (data not shown). Next, the capacity of RAW 264.7 cells to migrate across the uncoated membrane in transmigration assays was tested in the presence of breast cancer cell CM (Figure 4b). Our data clearly showed that CM derived from AnaR cells enhanced macrophage motility at a higher extent than CM derived from MCF-7 aro cells. This effect is tightly dependent on leptin signaling activation since AnaR cell-secreted factors did not induce any significant migratory effects when RAW 264.7 cells were treated with the peptide LDFI or when AnaR-CM immunodepleted of leptin (LepAb) was used as a chemoattractant (Figure 4c). As expected, the addition of leptin in MCF-7 aro cell-derived CM resulted in the induction of macrophage motility, which was specifically reversed by treatment of RAW 264.7 cells with the leptin antagonist peptide LDFI (Figure 4d). Therefore, the mechanisms underlying aggressive behavior of anastrozole resistant breast tumors seems to be not only intrinsic to cancer cells, but it may also rely on their ability to control macrophage migratory capability and their subsequent activation.

### 3.4. AnaR Cells Affect Motility of Macrophage through an Increase in CXCR4 expression

To gain insight into the molecular mechanisms responsible for the effects of AnaR cells on macrophage motility, quantitative transcriptome profiling of RAW 264.7 cells treated with MCF-7 aro- and AnaR cell-derived conditioned media was carried out by RNA sequencing analysis. This comparison allowed the identification of 1277 differentially expressed genes (FDR < 0.05), among which 73 were downregulated and 83 upregulated in response to AnaR-CM treatment when considering |1.5| fold as a cut-off. These genes were then subjected to Ingenuity Pathway Analysis (IPA) to rank enriched functions, and to calculate their relative activation z-score. In agreement with our previous results, the functional analysis revealed cellular movement among the most represented and upregulated function (activation z-score of 1.797, *p* = 1.97 × 10^−3^) (Table 1). Importantly, we have also evidenced that the most upregulated gene associated with AnaR-CM treatment was represented by CXCR4 (2.7 fold, *p* = 4.53 × 10^−21^), a well-known receptor involved in regulating cell movement [53,54,55,56].

**Figure 5:**
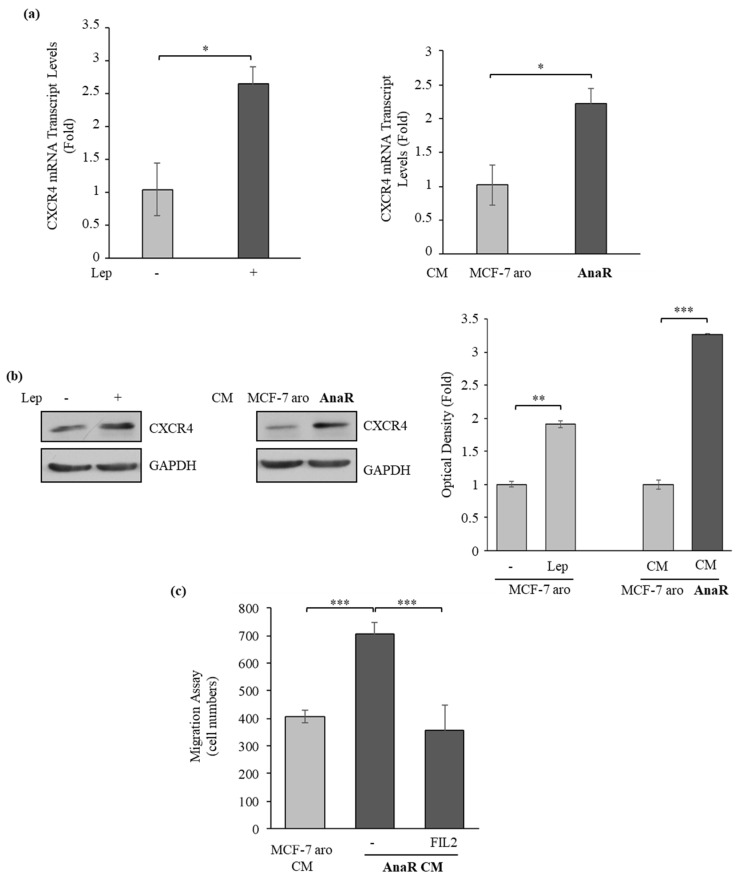
CXCR4 is involved in AnaR cell-mediated effects on macrophage motility. (**a**) Quantitative real-time PCR for CXCR4 mRNA expression in RAW 264.7 cells treated with vehicle (-), leptin (Lep 1000 ng/mL), conditioned medium (CM) derived from MCF-7 aro or from AnaR cells. (**b**) Immunoblotting for CXCR4 protein expression in RAW 264.7 cells treated as indicated. The histograms represent the mean ± S.D. of three separate experiments in which band intensities were evaluated in terms of optical density arbitrary units and expressed as fold over leptin or MCF-7 aro CM. (**c**) Transmigration assay in RAW 264.7 cells treated with vehicle (-) or the CXCR4 antagonist FIL2 using as a chemoattractant AnaR CM. MCF-7 aro CM as a chemoattractant was used as a control. * *p* < 0.05; ** *p* < 0.005; *** *p* < 0.0005.

To confirm the gene expression profile obtained in RNA sequencing analysis, we first compared mRNA expression of CXCR4 in RAW 264.7 cells treated with MCF-7 aro CM or AnaR CM. RAW 264.7 cells were also treated with leptin as a control. As shown in Figure 5a, a significant rise in mRNA levels of CXCR4 was detected in RAW 264.7 cells treated with leptin or AnaR-CM. This increase was then confirmed by evaluating protein levels by using immunoblotting (Figure 5b). To assess the role of CXCR4 in mediating AnaR-CM effects on macrophage recruitment, RAW 264.7 cells were treated with FIL2, a selective antagonist ligand of CXCR4 [57], and motility was assessed in the presence of AnaR CM. Interestingly, treatment with FIL2 significantly reversed the increase in macrophage migration induced by AnaR-CM (Figure 5c). Thus, AnaR cells may induce macrophage chemotaxis through increased CXCR4 expression.

Differentially expressed genes, as determined by RNA-Seq, were further analyzed with Ingenuity Pathway Analysis (IPA) software to outline the most enriched biological functions. The table shows the top five enriched terms corresponding to diseases/bio-functions along with the number of genes in the experimental dataset, *p*-value, and activation z-score.

### 3.5. Activated Macrophages Promote Growth and Migration of AnaR Cells

**Figure 6:**
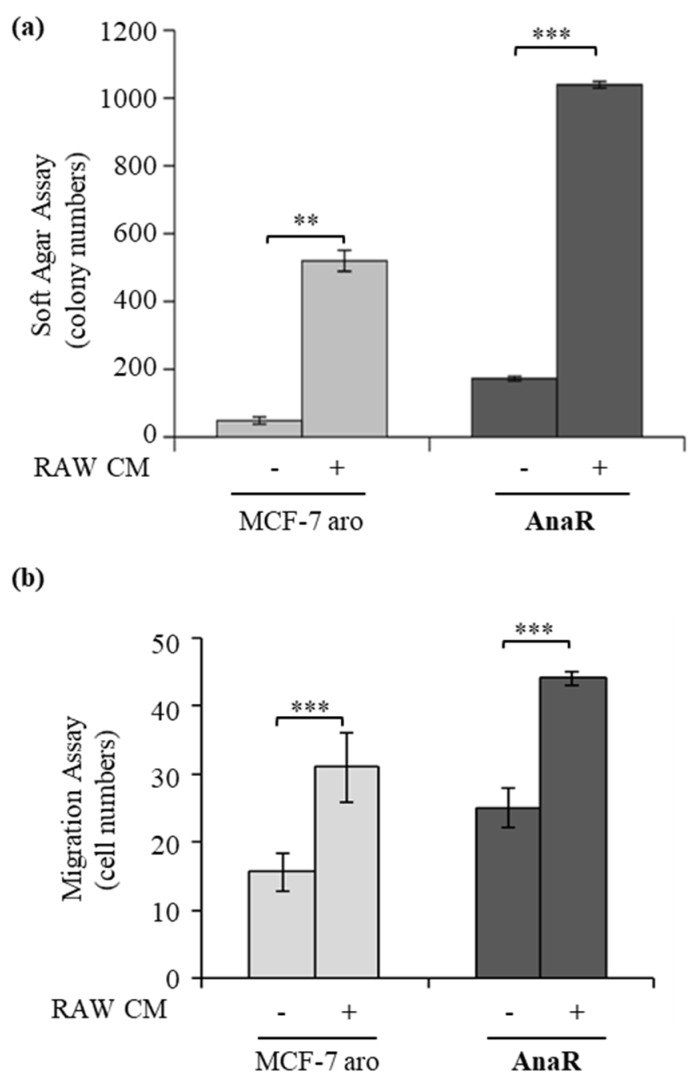
Activated macrophages support AnaR aggressive phenotype. (**a**) Soft agar growth assays in MCF-7aro and AnaR breast cancer cells treated control media (-) or conditioned media derived from activated RAW 264.7 cells (RAW CM). (**b**) Transmigration assay in MCF-7 aro and AnaR breast cancer cells using as chemoattractants control medium (-) or RAW CM. ** *p* < 0.005; *** *p* < 0.0005.

As a final step of this study, we further evaluated whether AnaR-activated macrophages may affect the proliferation and migration potential of breast cancer cells by co-culturing breast cancer cells with activated RAW 264.7 cells. As revealed by soft agar growth assay (Figure 6a), we found that incubation with conditioned media derived from activated RAW 264.7 cells resulted in an enhanced number of colonies of MCF-7 aro and AnaR cells, although with higher values in resistant cells. Accordingly, the capacity of AnaR cells to migrate across the uncoated membrane in transmigration assay was further increased in the presence of RAW 264.7 cell-derived CM compared to the effects observed in MCF-7 aro cells (Figure 6b). These data highlight the existence of an enhanced bidirectional crosstalk between anastrozole resistant breast cancer cells and macrophages that may be important to sustain AI^R^ tumor progression.

## 4. Discussion

Despite the clinical efficacy of the aromatase inhibitors in ERα-positive tumors, resistance often develops during treatment leading to disease progression. In the present study, we report a novel mechanism by which leptin signaling pathway impacts AI resistance, contributing to enhanced crosstalk between anastrozole-resistant breast cancer cells and macrophages within the tumor microenvironment.

The obesity-related adipokine leptin has been well characterized as a growth factor for breast cancer and has been recently proposed to decrease sensitivity to tamoxifen in vitro. It has been demonstrated that leptin treatment interferes with the antagonistic effects of tamoxifen in ERα-positive breast cancer cells [32,33] and the synergy between the leptin/Ob-R/STAT3 signaling pathway with the HER2 receptor induces tamoxifen resistance in breast cancer cells through differential regulation of apoptosis-related genes [35]. Interestingly, ObR knockdown significantly enhanced the inhibitory effects of tamoxifen on the proliferation and survival of tamoxifen-resistant cells [34]. We found that leptin may also have a role in AI resistance. Indeed, long-term anastrozole treatment of breast cancer cells induced leptin and ObR overexpression, which was associated with constitutive activation of leptin downstream effectors. Accordingly, inhibition of the leptin signaling pathway by using a specific peptide significantly reduced the growth and motility of AnaR breast cancer cells, highlighting the existence of an autocrine feedback loop that sustains the more aggressive behavior of AI resistant breast cancer cells. On the other hand, the increased expression of ObR in AnaR cells was also associated with enhanced sensitivity to exogenous leptin stimulation, as evidenced by the ability of increasing doses of leptin to stimulate anchorage-independent growth and migration at a greater extent in AnaR cells than in parental sensitive cells. These latter results suggest that the observed leptin-mediated impact in AnaR cells may be further enhanced in obese patients exhibiting higher circulating leptin levels. Thus, we can speculate that leptin might play a crucial role in the link between obesity and drug-resistant phenotypes in breast cancer.

An additional important factor increasing breast cancer risks in obesity is adipocyte-related inflammation, wherein the recruitment of macrophages to the tumor microenvironment represents a critical step [58]. In mouse models, different subpopulations of tumor-associated macrophages (TAMs) have been demonstrated to promote angiogenesis, tumor cell invasion, intravasation, and, at the metastatic site, tumor cell extravasation and persistent growth [58]. It is noteworthy that TAMs could be considered as alternatively activated M2 macrophages that are distinct from classically activated M1 macrophages and possess pro-tumoral functions. Clinical evidence has revealed a strong correlation between a high density of TAMs and poor prognosis in breast cancer [59]. In vivo experiments also demonstrated that adipose tissue within the mammary tumor microenvironment of obese mice exhibited higher numbers of macrophages and crown-like structures (CLS) than that of lean tumor-bearers [31]. Interestingly, leptin, through canonical ObR signaling activation, has been identified as a monocyte/macrophage chemoattractant and inducer of macrophage functions with potential relevance to their tissue accumulation and tumor-promoting effects [22,50,51,52]. Leptin seems to participate in the crosstalk between breast cancer cells and tumor-associated macrophages M2 by stimulating interleukin production to indirectly promote tumor breast cancer cell invasion and metastasis [51,52]. Moreover, leptin exposure during macrophage differentiation results in cells expressing M2 surface markers and high amounts of proinflammatory cytokines and specific chemokines, that contributes to the features observed in macrophages infiltrating the adipose tissue [60]. Here, we demonstrated that AnaR cells through leptin secretion promote an M2-like phenotype and increased macrophage motility. The potential chemotactic role of leptin secreted by AnaR is mediated by increased CXCR4 expression in macrophages, as evidenced by RNA sequencing analysis, real-time PCR, and immunoblotting. Accordingly, inhibition of CXCR4 signaling abrogated the effects of AnaR cell-derived conditioned media on macrophage motility. This is in line with evidence showing the multifaceted role of CXCR4 on regulating motility of different cell lines, including immune cells. Indeed, CXCR4 is expressed by leukocytes, macrophages, and hematopoietic stem/progenitor cells, and its signaling pathway may affect their chemiotaxis function [53,54,55,56]. It has been also demonstrated that during obesity, CXCR4 is involved in macrophage recruitment to the adipose tissue [61]. Additionally, hypoxia, a condition that has been associated with both obesity and leptin [62], increases CXCR4 expression on monocytes and macrophages, thereby inducing TAM trafficking and retention into the low oxygen tension tumor areas [63]. Thus, leptin-ObR-CXCR4 interplay may add another layer of complexity to the mechanisms underlying the deleterious effects of the breast cancer microenvironment in the resistant setting.

Examples of a bidirectional crosstalk between tumor cells and cells of the immune system has been reported recently, with tumor cells impacting the tumor microenvironment and immune cells inducing further amplification of tumor progression [64]. In the same way as AnaR cells affect macrophage phenotype, we found that, reciprocally, activated macrophages induced cancer cell growth and motility to a higher extent in AnaR cells, forming a positive feedback loop in coculture systems.

## 5. Conclusions

Previous studies have suggested that increased BMI (Body Mass Index) may represent an adverse prognostic factor in women receiving AIs (i.e., letrozole or anastrozole); for instance, the relative benefit of anastrozole vs. tamoxifen in the adjuvant or extended adjuvant setting may be lowered in obese women [11,65,66,67]. Accordingly, it has been recently demonstrated that mammary obese stromal cells induced increased aromatization-mediated ER transactivation and decreased sensitivity to anastrozole [68]. Therefore, obesity may be considered a risk factor for AI resistance; however, the molecular players underlying this link are still under investigation. Here, we provide evidence the obesity-related leptin, whose circulating levels increase proportionally to total adipose tissue mass, may be a critical factor promoting malignant AI^R^ growth in breast tissue. Our results reinforce the concept of leptin in the promotion of human malignancies and provide the rationale for additional studies aimed at verifying whether leptin signaling pathway may help to predict responses to endocrine therapies and could be exploited for the development of pinpoint alternative therapeutic strategies for the treatment of breast cancer patients, especially in the obese setting.

## Figures and Tables

**Table 1:** Top five enriched diseases and biological functions identified by Ingenuity Pathway Analysis (IPA) in RAW 264.7 cells treated with anastrozole resistant (AnaR) cell-derived conditioned media (CM) compared to cells treated with MCF-7 aro CM.

Categories	Diseases or Functions Annotation	*p*-Value	Activation z-score	Molecules	#Molecules
Cellular Movement	Invasion of tumor cell lines	1.97 × 10^−3^	1.797	ARRDC3, BCAR3, CXCR4, EDN1, FABP5, HIPK2, IL13RA2, LCN2, NDRG2, NOS2, SPP1, VEGFB	12
Cellular Movement, Hematological System Development and Function, Immune Cell Trafficking, Inflammatory Response	Chemotaxis of phagocytes	7.26 × 10^−4^	1.616	CSF1, CXCR4, IL1B, S100A8, SPP1, VEGFB	6
Cardiovascular System Development and Function, Cellular Development, Cellular Growth and Proliferation, Organismal Development, Tissue Development	Proliferation of endothelial cells	1.33 × 10^−5^	1.562	CSF1, EDN1, FABP4, GAS6, HIPK2, IL1B, PROCR, TNFSF15, VEGFB	9
Cellular Movement	Invasion of cells	1.67 × 10^−4^	1.487	ARRDC3, BCAR3, CLCA2, CXCR4, EDN1, FABP5, HIPK2, IER3, IL13RA2, IL1B, LCN2, NDRG2, NOS2, SPP1, VEGFB	15
Cellular Movement, Hematological System Development and Function, Immune Cell Trafficking, Inflammatory Response	Chemotaxis of myeloid cells	9.32 × 10^−4^	1.387	CSF1, CXCR4, IL1B, S100A8, SPP1, VEGFB	6

## Supplementary Materials

The following are available online at https://www.mdpi.com/2218-273X/10/4/543/s1, Table S1: Oligonucleotide primers used in this study.
