# Supplementary material for: Leptin Signaling Contributes to Aromatase Inhibitor Resistant Breast Cancer Cell Growth and Activation of Macrophages
Source: Biomolecules. 2020 Apr 3;10(4):543. doi: 10.3390/biom10040543 (PMC7226081; doi:10.3390/biom10040543)
Supplement: Supplementary file 1 [file biomolecules-10-00543-s001.pdf]

Table S1. Oligonucleotide primers used in this study.

Gene Name	*Gene Symbol*	*Species*		*Primer Sequences*
Leptin Receptor Long Isoform	*ObRl*	*Human*	Forward<br>Reverse	5’-GATAGAGGCCCAGGCATTTTTTA-3’<br>5’- CACCACTCTCTCTCTTTTTGATTGA-3’
Leptin Receptor Short Isoform	*ObRsh*	*Human*	Forward<br>Reverse	5’-ATTGTGCCAGTAATTATTTCCTCTTCC-3’<br>5’-CCACCATATGTTAACTCTCAGAAGTTCAA-3’
Leptin	*Ob*	*Human*	Forward<br>Reverse	5’-GAGACCTCCTCCATGTGCTG -3’<br>5’-TGAGCTCAGATATCGGGCTGAAC -3’
18s rRNA	*18s*	*Human*	Forward<br>Reverse	5’-CCCACTCCTCCACCTTTGAC-3’<br>5’-TGTTGCTGTAGCCAAATTCGTT-3’
Arginase-1	*ARG1*	*Mouse*	Forward<br>Reverse	5’-CTCCAAGCCAAAGTCCTTAGAG-3’<br>5’-AGGAGCTGTCATTAGGGACATC-3’
Interleukin-10	*IL10*	*Mouse*	Forward<br>Reverse	5’-CTGGACAACATACTGCTAACCG-3’<br>5’-GGGCATCACTTCTACCAGGTAA-3’
Nitric Oxide Synthase	*INOS*	*Mouse*	Forward<br>Reverse	5’-GTTCTCAGCCCAACAATACAAGA-3’<br>5’-GTGGACGGGTCGATGTCAC -3’
Interleukin-12	*IL12*	*Mouse*	Forward<br>Reverse	5’-TGGTTTGCCATCGTTTTGCTG-3’<br>5’-CAGGTGAGGTTCACTGTTTCT-3’
Chemokine (C-X-C motif) receptor 4	*CXCR4*	*Mouse*	Forward<br>Reverse	5’-GAAGTGGGGTCTGGAGACTAT -3’<br>5’-TTGCCGACTATGCCAGTCAAG-3’
Transforming growth factor beta	*TGF-β*	*Mouse*	Forward<br>Reverse	5’-ATCCTGTCCAAACTAAGGCTCG-3’<br>5’-ACCTCTTTAGCATAGTAGTCCGC-3’
SMAD family member 3	*SMAD3*	*Mouse*	Forward<br>Reverse	5’-GGATCCATGTCGTCCATCCTGCCC-3’<br>5’-GAATTCCTAAGACACACTGGAACAGCGGATG-3’
Glyceraldehyde 3-phosphate dehydrogenase	*GAPDH*	*Mouse*	Forward<br>Reverse	5’-CCCACTCCTCCACCTTTGAC-3’<br>5’-TGTTGCTGTAGCCAAATTCGTT-3’
